# Response to: Comment on “Sex Differences in the Association between Night Shift Work and the Risk of Cancers: A Meta-Analysis of 57 Articles”

**DOI:** 10.1155/2019/4391957

**Published:** 2019-07-07

**Authors:** Wen Liu, Zhonghan Zhou, Dahai Dong, Lijiang Sun, Guiming Zhang

**Affiliations:** Department of Urology, The Affiliated Hospital of Qingdao University, Qingdao, China

First of all, I would like to thank Professor Zhenyu Chen for his “Comment on “Sex Differences in the Association between Night Shift Work and the Risk of Cancers: A Meta-Analysis of 57 Articles”” [1]. The answers to the questions raised by Professor Chen are as follows.

In this paper, we conducted searches in strict accordance with PRISMA and the Cochrane handbook. We have indeed given a retrieval strategy in the original article: the search terms were “night shift work” or “rotating shift work” or “night work” or “shift work” and “carcinoma” or “neoplasm” or “tumor” or “cancer”, see Supplementary Search Strategy.

We stated in our article that we tested heterogeneity between studies by *I* ^2^ statistic with *I* ^2^ ≥ 50% indicating heterogeneity, and if no significant heterogeneity existed, a fixed effects model was adopted, otherwise a random effects model was used. For this question, we recalculated the data (Table 1) with a random effects model and verified that the outcomes in our article were correct [1], so we do not doubt the statistical methods in our study.

Due to the length of the article, the specific process of binary analysis was not presented. The binary analysis of dose-response relationship was performed before applying a generalized least-squares trend (GLST) model. The original data is shown in Supplementary Table 1. ORs and 95% CIs (the highest dose group compared with the reference dose group) were extracted to conduct binary analysis. The result of binary analysis was statistically significant (OR: 1.26; 95% CI: 1.13-1.40) (Figure 1), indicating that there was a positive association between night shift work and cancer. Therefore, the next step was to explore the dose-response relationship between night shift work and cancer. In addition, there are some meta-analysis articles which also analyzed dose-response relationship between night shift work and different cancer [2–5]. However, they did not mention the step of binary analysis in statistical methods, so we do not think that whether or not to mention binary analysis is the reason for questioning the dose-response relationship in our study.

We have analyzed the cause of publication bias and heterogeneity in our paper. First, as we have discussed in this paper, the contour-enhanced funnel plot and the trim and fill method were used together to analyze the cause of publication bias. The result showed that most of the filled studies were outside the 10% line, which indicated that the previously verified bias might be caused by heterogeneity, not the publication bias. Second, in the process of meta-analysis, a random effects model was used to minimize the influence of heterogeneity. Third, subgroup analyses and metaregression analyses were performed to assess the potential heterogeneity sources. Many subgroups, such as fixed shift, digestive system cancer, hematological system cancer, reproductive system cancer, and lung cancer, could decrease the value of *I* ^2^ and explain part of the heterogeneity (*P* > 0.05). As we have pointed out in the discussion, we attribute the remnant heterogeneity to inconsistent definition of work schedules, unclassified occupation based on population, ethnicity, and intrinsic defect of retrospective design; thus, further prospective study in a large-scale population should be performed to explore the relationship between night shift work and cancer. Fourth, leave-one-out analyses indicated a stable positive relationship between night shift work and the risk of cancer when the value of *I* ^2^ decreases to 29.8%. Therefore, we think that the conclusion of our study is credible and the closest to the truth so far.

In summary, we believe that the final conclusion of the paper after objective analysis is credible.

## Figures and Tables

**Figure 1:**
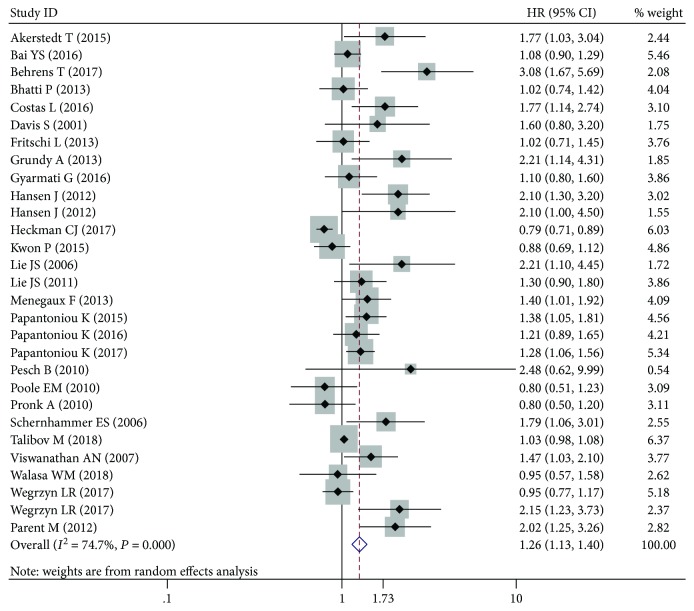
The outcome of binary analysis.

**Table 1:** The data for calculating sex differences in the association between night shift work and cancer risk.

Study	OR	LCI	UCI	Gender
Walasa WM (2018)	0.95	0.57	1.58	Female
Talibov M (2018)	1.03	0.98	1.08	Female
Papantoniou K (2016)	1.21	0.89	1.65	Female
Wang P (2015)	1.34	1.05	1.72	Female
Li WJ (2015)	0.73	0.66	0.82	Female
Datta K (2014)	1.51	0.27	8.52	Female
Rabstein S (2013)	1.01	0.68	1.5	Female
Fritschi L (2013)	1.02	0.71	1.45	Female
Menegaux F (2013)	1.4	1.01	1.92	Female
Grundy A (2013)	2.21	1.14	4.31	Female
Bhatti P (2013)	1.02	0.74	1.42	Female
Hansen J (2012)	2.1	1.3	3.2	Female
Hansen J (2012)	2.1	1	4.5	Female
Lie JS (2011)	1.3	0.9	1.8	Female
Lie JS (2006)	2.21	1.1	4.45	Female
Pesch B (2010)	2.48	0.62	9.99	Female
Hansen J (2001)	1.5	1.3	1.7	Female
Truong (2014)	1.32	1.02	1.72	Female
Kwon P (2015)	0.88	0.69	1.12	Female
Davis S (2001)	1.6	0.8	3.2	Female
Leary ES (2006)	1.04	0.79	1.38	Female
Devore EE (2017)	0.96	0.83	1.11	Female
Knutsson A (2013)	2.02	1.03	3.95	Female
Carter BD (2014)	1.27	1.03	1.56	Female
Poole EM (2010)	0.8	0.51	1.23	Female
Viswanathan AN (2007)	1.47	1.03	2.1	Female
Akerstedt T (2015)	1.77	1.03	3.04	Female
Koppes LLJ (2014)	0.87	0.72	1.05	Female
Natti J (2012)	2.82	1.2	6.65	Female
Schernhammer ES (2006)	1.79	1.06	3.01	Female
Pronk A (2010)	0.8	0.5	1.2	Female
Schernhammer ES (2003)	1.35	1.03	1.77	Female
Vistisen HT (2017)	0.9	0.8	1.01	Female
Schernhammer ES (2013)	1.28	1.07	1.53	Female
Gu FY (2015)	1.08	0.98	1.19	Female
Lahti TA (2008)	1.02	0.94	1.12	Female
Bai YS (2016)	0.9	0.66	1.23	Female
Travis RC (2016)	1	0.92	1.08	Female
Wegrzyn LR (2017)	0.95	0.77	1.17	Female
Wegrzyn LR (2017)	2.15	1.23	3.73	Female
Heckman CJ (2017)	0.79	0.71	0.89	Female
Jorgensen JT (2017)	0.91	0.77	1.08	Female
Talibov M (2018)	1.03	0.98	1.09	Male
Tse LA (2017)	1.76	1.07	2.89	Male
Papantoniou K (2015)	1.38	1.05	1.81	Male
Parent M (2012)	2.02	1.25	3.26	Male
Natti J (2012)	1.78	0.8	4	Male
Lahti TA (2008)	1.1	1.03	1.19	Male
Bai YS (2016)	1.27	1.01	1.59	Male
Akerstedt T (2017)	0.91	0.74	1.12	Male
Dickerman BA (2016)	1	0.7	1.2	Male
Lin YS (2015)	1.43	0.78	2.63	Male
Hammer GP (2015)	0.93	0.73	1.18	Male
Gapstur SM (2014)	1.08	0.95	1.22	Male
Kubo T (2011)	1.79	0.57	5.68	Male
Behrens T (2017)	3.08	1.67	5.69	Male
Kubo T (2006)	3	1.2	7.7	Male
Lin YS (2013)	0.83	0.43	1.6	Male
Yong M (2014)	1.04	0.89	1.21	Male

Abbreviations: OR: odds ratio; LCI: lower confidence interval; UCI: upper confidence interval.

## Supplementary Materials

Supplementary Materials Supplementary Search Strategy and Supplementary Table 1.

## References

[1] Sun P., Bi M., Su Y., Chen Z. (2019). Comment on “Sex differences in the association between night shift work and the risk of cancers: a meta-analysis of 57 articles”. *Disease Markers*.

[2] Gan Y., Li L., Zhang L. (2018). Association between shift work and risk of prostate cancer: a systematic review and meta-analysis of observational studies. *Carcinogenesis*.

[3] Rao D., Yu H., Bai Y., Zheng X., Xie L. (2015). Does night-shift work increase the risk of prostate cancer? A systematic review and meta-analysis. *OncoTargets and Therapy*.

[4] Wang X., Ji A., Zhu Y. (2015). A meta-analysis including dose-response relationship between night shift work and the risk of colorectal cancer. *Oncotarget*.

[5] Yuan X., Zhu C., Wang M., Mo F., Du W., Ma X. (2018). Night shift work increases the risks of multiple primary cancers in women: a systematic review and meta-analysis of 61 articles. *Cancer Epidemiology Biomarkers & Prevention*.

