# Supplementary material for: Response to: Comment on “Sex Differences in the Association between Night Shift Work and the Risk of Cancers: A Meta-Analysis of 57 Articles”
Source: Dis Markers. 2019 Jul 7;2019:4391957. doi: 10.1155/2019/4391957 (PMC6642780; doi:10.1155/2019/4391957)
Supplement: Supplementary Materials — Supplementary Search Strategy and Supplementary Table 1. [file 4391957.f1.pdf]

Supplementary Search Strategy

Search items in pubmed:

#1 "Neoplasms"[Mesh]

#2 neoplasm [Title/Abstract]

#3 neoplasms [Title/Abstract]

#4 carcinoma [Title/Abstract]

#5 carcinomas [Title/Abstract]

#6 tumor [Title/Abstract]

#7 tumors [Title/Abstract]

#8 tumour [Title/Abstract]

#9 tumours [Title/Abstract]

#10 cancer [Title/Abstract]

#11 cancers [Title/Abstract]

#12 #1 OR #2 OR #3 OR #4 OR #5 OR #6 OR #7 OR #8 OR #9 OR #10 OR #11

#13 "Shift Work Schedule"[Mesh]

#14 "Shift Work Schedule"[Title/Abstract]

#15 "night shift work" [Title/Abstract]

#16 "night work"[Title/Abstract]

#17 "rotating shift work"[Title/Abstract]

#18 "shift work" [Title/Abstract]

#19 #13 OR #14 OR #15 OR #16 OR #17 OR #18

#20 #12 AND #19

Supplementary Table 1: The extracted data for calculating dose-response relationship.

Study	Number	Type	Dose（year）	Cases	Total Subjects	OR	LCI	UCI
Walasa WM(2018)	1	1	0	277	605	1	1	1
	1	1	4	40	81	1.17	0.73	1.88
	1	1	9	33	74	0.95	0.57	1.58
TalibovM(2018)	2	1	0	129,503	657970	1	1	1
	2	1	6	20,806	126987	0.977	0.961	0.993
	2	1	15	2,795	16400	1.024	0.983	1.068
	2	1	24	2011	11719	1.033	0.984	1.084
Papantoniou K(2016)	3	1	0	1438	2980	1	1	1
	3	1	3	67	125	1.21	0.83	1.76
	3	1	10	103	188	1.13	0.83	1.53
	3	1	18	97	188	1.21	0.89	1.65
Papantoniou K(2017)	4	1	0	1071	2432	1	1	1
	4	1	7.5	147	282	1.19	0.95	1.49
	4	1	18	274	361	1.28	1.06	1.56
Gyarmati G(2016)	5	1	0	278	2202	1	1	1
	5	1	5.5	28	219	1.1	0.7	1.6
	5	1	15.5	21	141	1.1	0.7	1.9
	5	1	24	47	293	1.1	0.8	1.6
Costas L(2016)	6	1	0	225	1468	1	1	1
	6	1	3	22	138	0.86	0.52	1.43
	6	1	13	17	127	0.65	0.37	1.13
	6	1	24	39	152	1.77	1.14	2.74
Papantoniou K(2015)	7	1	0	733	1717	1	1	1
	7	1	5.5	128	273	1.1	0.83	1.45
	7	1	18.5	92	222	0.94	0.69	1.27
	7	1	33.6	138	266	1.38	1.05	1.81
Fritschi L(2013)	8	1	0	914	2318	1	1	1
	8	1	5.5	164	363	1.25	1	1.56
	8	1	15.5	71	169	1.09	0.79	1.5
	8	1	24	53	137	1.02	0.71	1.45
Menegaux F(2013)	9	1	0	1068	1170	1	1	1
	9	1	2.3	66	69	1.12	0.78	1.6
	9	1	5.4	98	78	1.4	1.01	1.92
Grundy A(2013)	10	1	0	751	1524	1	1	1
	10	1	7.5	283	595	0.95	0.79	1.16
	10	1	22.5	72	153	0.93	0.67	1.3
	10	1	36	28	41	2.21	1.14	4.31
Bhatti P(2013)	11	1	0	808	2228	1	1	1
	11	1	0.7	55	152	1.03	0.72	1.47
	11	1	2	75	196	1.13	0.82	1.54
	11	1	5	94	179	1.95	1.41	2.68
	11	1	8.4	68	176	1.02	0.74	1.42
Hansen J(2012)	12	1	0	37	289	1	1	1
	12	1	3	55	283	1.5	0.99	2.5
	12	1	7.5	70	265	2.3	1.4	3.5
	12	1	15	66	302	1.9	1.1	2.8
	12	1	24	39	163	2.1	1.3	3.2
Parent M(2012)	13	1	0	74	512	1	1	1
	13	1	2.5	423	2761	2.201	1.543	3.14
	13	1	7.5	170	593	1.676	1.032	2.722
	13	1	12	204	374	2.016	1.246	3.261
Hansen J(2012)	14	1	0	88	449	1	1	1

	14	1	3.5	13	80	0.9	0.4	1.7
	14	1	11.5	18	66	1.7	0.9	3.2
	14	1	18	12	41	2.1	1	4.5
Lie JS(2011)	15	1	0	102	250	1	1	1
	15	1	6.5	410	933	1.2	0.9	1.5
	15	1	14.4	187	411	1.3	0.9	1.8
Lie JS(2006)	16	1	0	50	265	1	1	1
	16	1	7.5	362	1873	0.95	0.67	1.33
	16	1	22.5	101	460	1.29	0.82	2.02
	16	1	36	24	82	2.21	1.1	4.45
Pesch B(2010)	17	1	0	698	1438	1	1	1
	17	1	2.5	15	40	0.65	0.28	1.48
	17	1	7.5	11	23	0.93	0,31	2.82
	17	1	15	10	21	0.83	0.27	2.6
	17	1	24	12	17	2.48	0.62	9.99
Kwon P(2015)	18	1	0	438	1284	1	1	1
	18	1	8.5	356	1165	0.82	0.68	0.98
	18	1	21	259	806	0.89	0.72	1.09
	18	1	27.8	223	610	0.9	0.72	1.12
	18	1	36.7	175	606	0.88	0.69	1.12
Davis S(2001)	19	1	0	733	1451	1	1	1
	19	1	1.5	15	26	1.4	0.6	3.2
	19	1	3.6	19	33	1.6	0.8	3.2
Poole EM(2010)	20	2	0	270	1056596	1	1	1
	20	2	2	197	862237	1.07	0.89	1.29
	20	2	4.5	115	569716	0.9	0.72	1.13
	20	2	8	51	253098	0.92	0.68	1.25
	20	2	12	39	125268	1.14	0.81	1.6
	20	2	17.5	24	53850	1.28	0.84	1.94
	20	2	24	22	53907	0.8	0.51	1.23
Viswanathan AN(2007)	21	2	0	210	298283	1	1	1
	21	2	5.5	224	343742	0.89	0.74	1.08
	21	2	15	43	49099	1.06	0.76	1.49
	21	2	24	38	29574	1.47	1.03	2.1
Akerstedt T(2015)	22	3	0	354	9674	1	1	1
	22	3	3.5	57	1671	0.93	0.66	1.31
	22	3	8.5	16	639	0.79	0.45	1.38
	22	3	16	18	614	0.8	0.45	1.42
	22	3	33.5	18	305	1.77	1.03	3.04
Schernhammer ES(2006)	23	2	0	441	426119	1	1	1
	23	2	5.5	816	809374	0.98	0.87	1.1
	23	2	15	80	72829	0.91	0.72	1.16
	23	2	24	15	4881	1.79	1.06	3.01
PronkA(2010)	24	3	0	276	51238	1	1	1
	24	3	2.5	25	6055	0.9	0.6	1.3
	24	3	11	29	6459	0.9	0.6	1.4
	24	3	20.4	19	5720	0.8	0.5	1.2
Bai YS(2016)	25	3	0	822	16690	1	1	1
	25	3	5	108	1988	1.19	0.97	1.46
	25	3	15	140	3039	1.06	0.88	1.27
	25	3	24	152	3069	1.08	0.9	1.29
Wegrzyn LR(2017)	26	2	0	2382	640594	1	1	1
	26	2	7.5	3162	817778	1.01	0.96	1.07
	26	2	22.5	331	84887	1.06	0.94	1.19

	26	2	36	96	25178	0.95	0.77	1.17
Wegrzyn LR(2017)	27	2	0	1318	978847	1	1	1
	27	2	5.5	2071	1475921	1.05	0.98	1.13
	27	2	15	168	112752	1	0.85	1.17
	27	2	24	13	3335	2.15	1.23	3.73
Heckman CJ(2017)	28	2	0	1506	558227	1	1	1
	28	2	1	1326	529084	0.929	0.863	1
	28	2	4	1151	469373	0.909	0.842	0.982
	28	2	8	478	218168	0.812	0.733	0.9
	28	2	12	393	183378	0.794	0.711	0.888
Behrens T(2017)	29	2	0	38	12168	1	1	1
	29	2	5.5	13	2500	1.87	0.99	3.55
	29	2	15	8	1312	2.18	1.01	4.72
	29	2	24	17	2245	3.08	1.67	5.69

Abbreviations: OR: odds ratio; LCI: lower confidence interval; UCI: upper confidence interval.
